# KY19382 Accelerates Cutaneous Wound Healing via Activation of the Wnt/β-Catenin Signaling Pathway

**DOI:** 10.3390/ijms241411742

**Published:** 2023-07-21

**Authors:** Minguen Yoon, Eunhwan Kim, Seol Hwa Seo, Geon-Uk Kim, Kang-Yell Choi

**Affiliations:** 1 Department of Biotechnology, College of Life Science and Biotechnology, Yonsei University, Seoul 03722, Republic of Korea; kavelon@nate.com (M.Y.); glowlight18@outlook.com (E.K.); seolhwa87@hotmail.com (S.H.S.); kwin0125@naver.com (G.-U.K.); 2 CK Regeon Inc., Seoul 03722, Republic of Korea

**Keywords:** human keratinocyte, human dermal fibroblast, cutaneous wound healing, Wnt/β-catenin signaling pathway, regeneration

## Abstract

The Wnt/β-catenin signaling pathway plays important roles in the multi-phases of wound healing: homeostasis, inflammation, proliferative, and remodeling phases. However, there are no clinically available therapeutic agents targeting the Wnt/β-catenin pathway. In this study, we tested the effect of 5, 6-dichloroindirubin-3′-methoxime (KY19382), a small molecule that activates the Wnt/β-catenin pathway via interference with the function of the negative feedback regulator CXXC5, on cutaneous wound healing. KY19382 significantly enhanced cell migration of human keratinocytes and dermal fibroblasts with increased levels of β-catenin, phalloidin, Keratin 14, proliferating cell nuclear antigen (PCNA), Collagen I, and alpha-smooth muscle actin (α-SMA) by activating the Wnt/β-catenin signaling pathway without causing significant cytotoxicity. In addition, levels of Collagen I, Keratin 14, PCNA, and stem cell markers were significantly increased by KY19382 in a cutaneous murine wound healing model. Moreover, KY19382 treatment accelerated re-epithelialization and neo-epidermis formation with collagen deposition and stem cell activation at an early stage of cutaneous wound healing. Overall, KY19382 accelerates wound healing via activating the Wnt/β-catenin pathway, and may have the potential to be used for the development of a new wound healing agent.

## 1. Introduction

Wound healing is a dynamic process orchestrated by interactions among various cells, cytokines, and growth factors [1]. These interactions restore the function and structural damage of tissues through the homeostasis, inflammatory, proliferative, and remodeling phases [2]. Failure of the delegate processes may cause a delay in wound healing and abnormal scar formation [3].

Due to the increase in elderly population, the incidence of chronic wounds is rising, and the global market for wound healing agents is consistently growing [4].

Traditionally, most skin wounds are handled by dressings and antibiotics to prevent further infection [5]. More recently, wound healing agents containing growth factors are used [6]. However, antibiotic therapies often result in functional impairments that cause defects in skin structures, and therapies using growth factors are practically limited owing to their high cost, poor efficacy, and low delivery rate [5,7]. Stem cell therapy is considered a regenerative approach but has limitations in quality control safety, cost, and administration [8,9].

Therefore, advanced wound healing agents that can induce the regeneration of damaged tissues are needed to overcome problems of current therapies.

In recent years, advancements have been made in understanding the relationship between the wound healing process and signaling mechanisms [7]. In particular, understanding the signaling pathways involved in keratinocyte migration, extracellular matrix (ECM) formation, and collagen production is useful for the development of advanced therapeutic agents for the treatment of acute and chronic wounds [10]. Among the signaling pathways, the Wnt/β-catenin signaling pathway is considered a major player in cutaneous wound healing [11,12]. The Wnt/β-catenin pathway regulates early skin development, determining the proliferation and fate of progenitor cells [13,14]. The Wnt/β-catenin pathway plays a crucial role in tissue regeneration involving adult stem cells during wound healing [15,16]. Upregulation of the Wnt/β-catenin pathway induces migration, proliferation, and differentiation of dermal fibroblasts [17,18]. Moreover, the Wnt/β-catenin pathway is involved in multiple stages of wound healing [2] and cooperates with other diverse signaling pathways during the wound healing process [19,20,21]. Therefore, the Wnt/β-catenin pathway could be an innovative target for the development of wound healing agents.

Currently, several activators of the Wnt/β-catenin pathway have been characterized for their effect on enhancing wound healing [22,23,24].

In our previous study, we discovered that CXXC-type zinc finger protein 5 (CXXC5), a negative regulator of the Wnt/β-catenin pathway, is a major factor that negatively regulates cutaneous wound healing [25]. Therefore, we previously developed the protein transduction domain-fused Dvl binding motif (PTD-DBM), a peptide blocking CXXC5 function via interference of the CXXC5–Dvl protein–protein interaction (PPI), as a wound healing agent and its effectiveness was further enhanced by co-treatment with valproic acid, a direct activator of Wnt/β-catenin [25]. The PTD-DBM peptide enhanced the wound healing process, but also promoted regenerative hair growth via activation of the Wnt/β-catenin pathway [26]. Nevertheless, PTD-DBM is limited in its routine application due to the limitation of peptide agents such as stability and cost, etc.

We recently screened and obtained small molecules which mimic the function of PTD-DBM by development of an in vitro high-throughput screening system monitoring the CXXC5–Dvl PPI and subsequent characterization of Wnt/β-catenin signaling activation [27]. KY19382, an indirubin derivate, activates the Wnt/β-catenin pathway by targeting both glycogen synthase kinase-3 beta (GSK3β) and CXXC5–Dvl PPI and effectively induced regenerative hair growth as well as the regrowth of hair by strong activation of Wnt/β-catenin signaling [28]. However, the activity of KY19382 in wound healing has not been discovered.

In this study, we tested the effect of KY19382 on acute wound healing by using both in vitro and in vivo analyses. KY19382 effectively accelerated in vitro wound healing with enhancement of the migration of the keratinocytes and fibroblasts by activating the Wnt/β-catenin signaling pathway. Furthermore, KY19382 accelerated cutaneous wound healing by stimulation of collagen deposition, re-epithelialization, neo-epidermis formation, and activation of stem cells by activating the Wnt/β-catenin pathway.

Collectively, KY19382 accelerates cutaneous wound healing by activating the Wnt/β-catenin pathway, and it could be used as a therapeutic wound healing agent.

## 2. Results

### 2.1. KY19382 Enhances the Migration of Human Keratinocytes and Dermal Fibroblasts via Activation of the Wnt/β-Catenin Signaling Pathway

As previously known, we confirmed that KY19382 significantly activated Wnt/β-catenin signaling, as revealed by a dose-dependent increment of the TOPflash reporter activity [27] (Figure S1).

We tested the effect of KY19382 on the migration of both human keratinocytes and dermal fibroblasts, which are known to play important roles in cutaneous wound healing and skin homeostasis [29,30]. The optimal concentration of KY19382 in wound healing of both human keratinocytes and dermal fibroblasts was determined as 1 μM, as confirmed by in vitro cell migration assay (Figure S2). No significant cellular toxicity was observed, as shown by the measurement of viable cells for the keratinocytes and dermal fibroblasts as well as the fragile primary neural stem cells (Figure S3). Thus, 0.1 and 1 μM KY19382 were used for subsequent in vitro studies and 10 μM PTD-DBM was used as a positive control [25].

**Figure 1:**
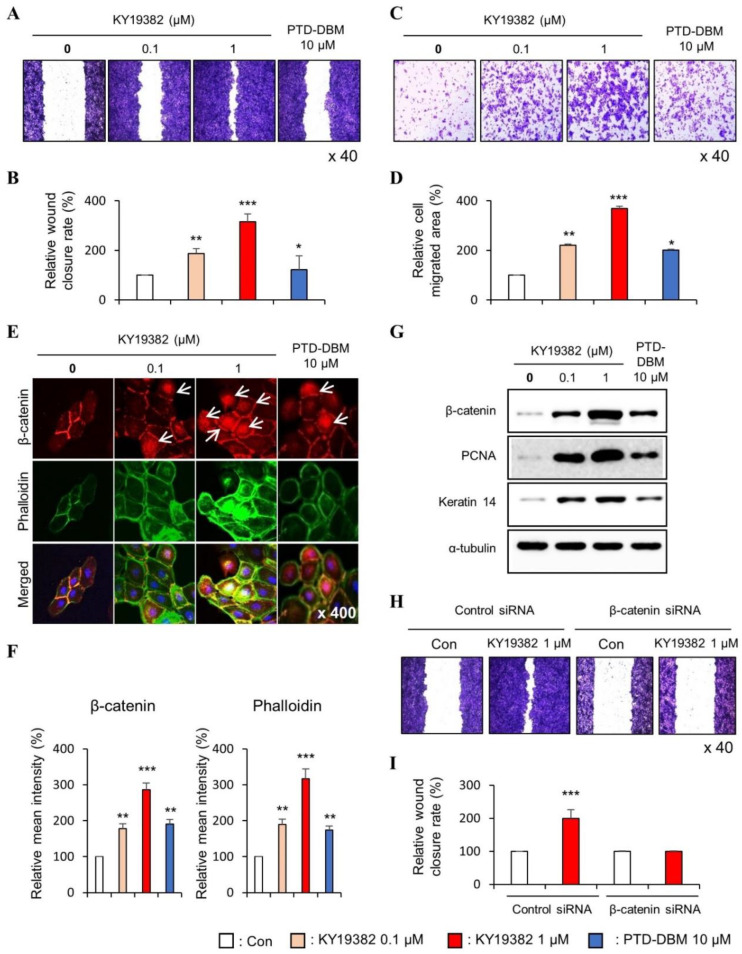
Effects of KY19382 on migration of human keratinocytes. (**A**–**G**) HaCaT keratinocytes were treated with vehicle (0.1% [*v*/*v*] DMSO), KY19382 (0.1, 1 μM), or PTD-DBM (10 μM) for 24 h. (**A**–**D**) The in vitro wound healing and transwell assays were performed as described in Materials and Methods. (**A**) Representative images of in vitro wound healing assay. (**B**) Quantitative measurement of relative wound closure rate in Figure 1A. (**C**) Representative images of transwell assay. (**D**) Quantitative measurement of the area of migrated cells in Figure 1C. (**E**) ICC staining for β-catenin and total phalloidin. Nuclei were counterstained with DAPI. Arrows indicate nucleus-localized β-catenin. (**F**) Quantitative measurements of intensities of β-catenin and phalloidin in Figure 1E (*n* = 12 taken in three different images). (**G**) Immunoblotting analysis data. (**H**,**I**) HaCaT keratinocytes were transfected with β-catenin siRNA or negative control for 12 h. After transfection, HaCaT keratinocytes were treated with vehicle (0.1% DMSO) or KY19382 1 μM for 24 h. (**H**) Representative images of in vitro wound healing assay of transfected keratinocytes. (**I**) Quantitative measurement of wound closure rate in Figure 1H. Original magnification: (**A**,**C**,**H**), ×40; (**E**), ×400. Values are expressed as means ± SEM. *, *p* < 0.05; **, *p* < 0.005; ***, *p* < 0.0005, significantly different from vehicle, control, or as indicated.

**Figure 2:**
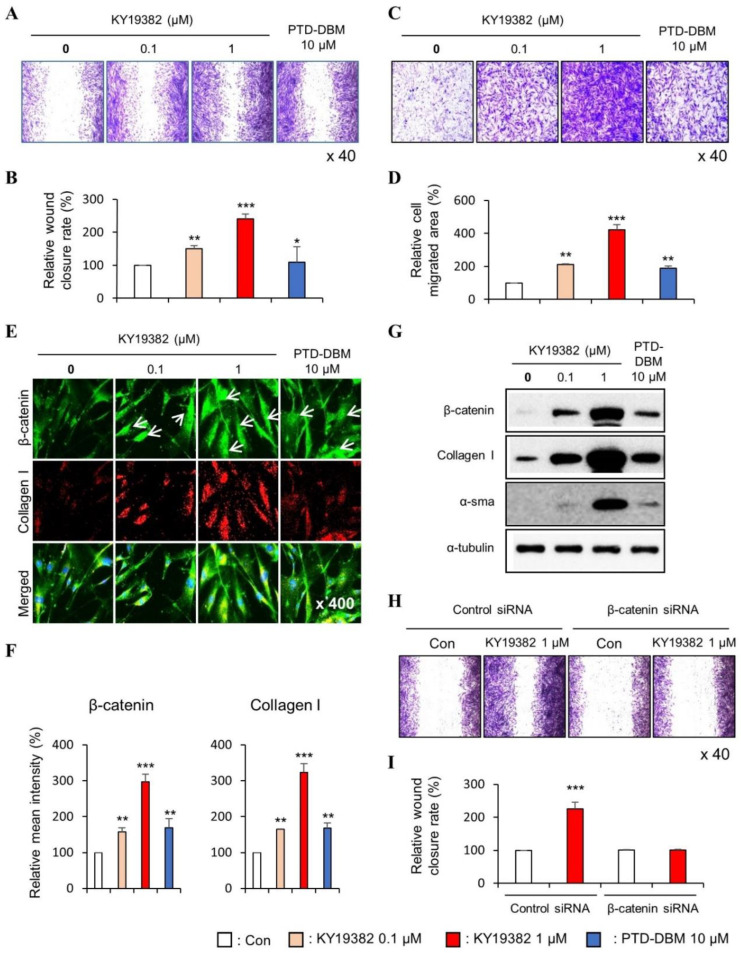
Effects of KY19382 on migration of human dermal fibroblasts. (**A**–**G**) Human dermal fibroblasts were treated with vehicle (0.1% DMSO), KY19382 (0.1, 1 μM), or PTD-DBM (10 μM) for 24 h. (**A**–**D**) The in vitro wound healing and transwell assays were performed as described in Materials and Methods. (**A**) Representative images of in vitro scratch assay. (**B**) Quantitative measurement of relative scratch closing rate in Figure 2A. (**C**) Representative images of transwell assay. (**D**) Quantitative measurement of the area of migrated cells in Figure 2C. (**E**) ICC staining for β-catenin and Collagen I. Nuclei were counterstained with DAPI. Arrows indicate nucleus-localized β-catenin. (**F**) Quantitative measurements of intensities of β-catenin and Collagen I in Figure 2E (*n* = 10 taken in three different images). (**G**) Immunoblotting analysis data. (**H**,**I**) Fibroblasts were transfected with β-catenin siRNA or negative control for 12 h. After transfection, fibroblasts were treated with vehicle (0.1% DMSO) or KY19382 1 μM for 24 h. (**H**) Representative images of in vitro wound healing assay of transfected fibroblasts. (**I**) Quantitative measurement of wound closure rate in Figure 2H. Original magnification: (**A**,**C**,**H**), ×40; (**E**), ×400. Values are expressed as means ± SEM. *, *p* < 0.05; **, *p* < 0.005; ***, *p* < 0.0005, significantly different from vehicle, control, or as indicated.

To confirm the role of KY19382 in cell migration and proliferation, we performed in vitro wound healing assay and transwell assay which can define both properties. The migration of both keratinocytes and fibroblasts were increased dose-dependently by KY19382 treatment, as shown by both in vitro wound healing and transwell assays (Figure 1A–D and Figure 2A–D). To identify whether KY19382 accelerates in vitro wound healing by activation of Wnt/β-catenin signaling, we checked the level of β-catenin by using immunocytochemistry (ICC) analyses in human keratinocytes and fibroblasts. Upon treatment with KY19382, active β-catenin, which is nuclear-localized, was dose-dependently increased with increments of phalloidin, a cortical networking F-actin marker, and Collagen I in human keratinocytes and fibroblasts (Figure 1E,F and Figure 2E,F). The effects of KY19382 in the Wnt/β-catenin signaling activation and phalloidin increment were more significant than that of PTD-DBM (Figure 1E,F and Figure 2E,F). As confirmed by immunoblot analyses, KY19382 treatment dose-dependently increased the levels of β-catenin, PCNA, and Keratin 14, a marker for re-epithelialization, and terminally differentiated keratinocytes, in human keratinocytes (Figure 1G). KY19382 treatment also dose-dependently increased Collagen I, a marker for myo-fibroblast differentiation, and α-SMA, a marker for fibroblast contractility, in human dermal fibroblasts (Figure 2G). These KY19382 effects in cell migration were acquired through activation of Wnt/β-catenin signaling, as confirmed by the abolishment of the drug effect by β-catenin knockdown (Figure 1H,I and Figure 2H,I).

Collectively, these results indicate that KY19382 stimulates in vitro wound healing of keratinocytes and fibroblasts by activating the Wnt/β-catenin signaling pathway.

### 2.2. KY19382 Accelerates Cutaneous Wound Healing In Vivo with Activation of the Wnt/β-Catenin Pathway

**Figure 3:**
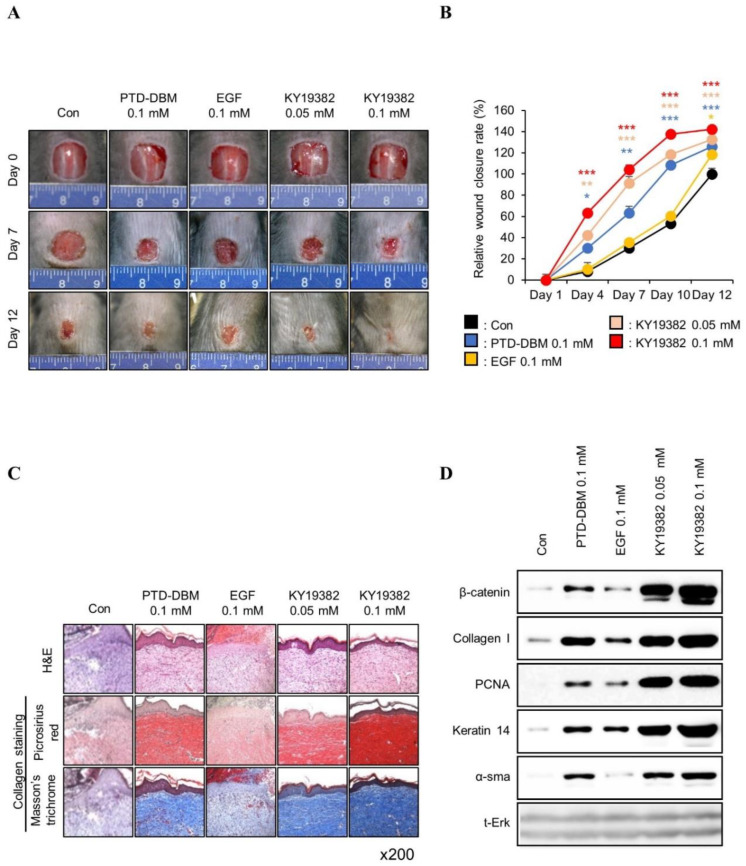
Effects of KY19382 on cutaneous wound healing. Full-thickness wounds (diameter = 1.0 cm) were made on the back of 8-week-old male C3H mice, and vehicle, KY19382 (0.05, 0.1 mM), PTD-DBM (0.1 mM), or EGF (0.1 mM) were topically applied daily on the wounds up to 12 d (*n* = 6 mice/group). Mice were sacrificed and wounded tissues excised at 12 d post wounding were subjected to histochemical analyses. (**A**) Representative gross images of wounds at 0, 7, or 12 d. (**B**) Relative wound closure rates were quantified as percentage of wound closure and closure rates of the control group were considered as 100. Wound sizes were measured every 1, 4, 7, 10, and 12 d after creating the wound. (**C**) Representative images of Masson’s trichrome and picrosirius red staining of wounded tissues 12 d post wounding (*n* = 3 independent experiments). (**D**) Immunoblotting analysis data for wounded tissue extracts obtained 12 d post wounding. Original magnification: (**C**), ×200. Values are expressed as means ± SEM. *, *p* < 0.05; **, *p* < 0.005; ***, *p* < 0.0005, significantly different from vehicle, control, or as indicated.

To determine the effect of KY19382 on in vivo cutaneous wound healing, we performed an in vivo wound healing assay using KY19382 (0.05 or 0.1 mM) with positive controls EGF [31] (0.1 M) and PTD-DBM (0.1 mM). The wound closure rate was increased dose-dependently with enhancement of re-epithelialization following treatment with KY19382 (Figure 3A,B). Both re-epithelialization and wound healing were mostly completed after 12 d of treatment with 0.1 mM KY19382, and its effectiveness was higher than that of EGF (Figure 3A,B). KY19382 dose-dependently increased collagen deposition, a hallmark of wound healing, in the dermal layer of wounded tissues, as shown by picrosirius red and Masson’s trichrome collagen staining results (Figure 3C). The effect of 0.1 mM KY19382 on collagen deposition was much higher than that of 0.1 mM PTD DBMP or 0.1 mM EGF (Figure 3C). KY19382 treatment significantly increased the protein levels of Collagen I, Keratin 14, PCNA, and α-SMA with activation of β-catenin, as confirmed by immunoblotting analyses of wound tissues harvested on 12 d (Figure 3D).

**Figure 4:**
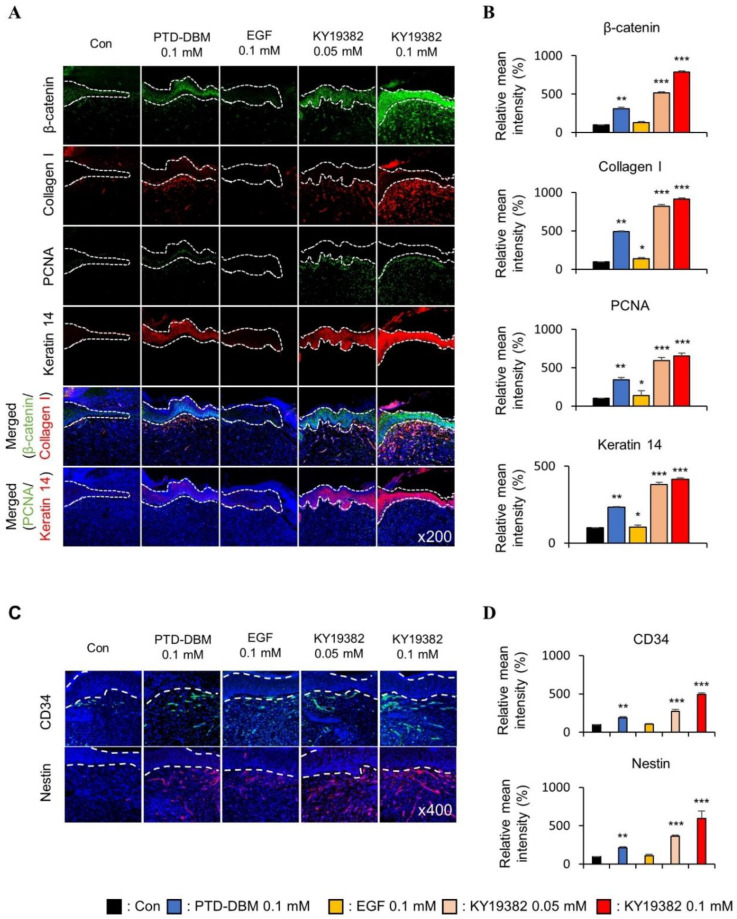
Effects of KY19382 in wound healing and stem cell markers. Wounded tissues excised from 12 d post wounding of Figure 3 were subjected to IHC analyses. (**A**) Representative images for IHC analyses of β-catenin, Collagen I, PCNA, and Keratin 14 at wounds 12 d post wounding. Dashed lines demarcate the epidermis boundary (*n* = 3, independent experiments). (**B**) Mean intensity values for Figure 4A (*n* = 3, independent experiments). (**C**) Representative images for IHC analyses of CD34 and Nestin at wounds 12 d post wounding. Dashed lines demarcate the epidermis boundary (*n* = 3, independent experiments). (**D**) Mean intensity values for Figure 4C (*n* = 3 independent experiments). Original magnification: (**A**), ×200; (**C**), ×400. Values are expressed as means ± SEM. *, *p* < 0.05; **, *p* < 0.005; ***, *p* < 0.0005, significantly different from vehicle, control, or as indicated.

To further characterize the wound healing effects of KY19382 related to Wnt/β-catenin signaling, immunohistochemical (IHC) analyses were performed with the wound tissues harvested on 12 d. The level of β-catenin was increased in both keratinocytes and fibroblasts of skin tissues after treatment with KY19382, and the level was much more significant than PTD-DBM (Figure 4A,B). By contrast, no increment of β-catenin was observed by EGF treatment (Figure 4A,B). The expression level of Collagen I was synergistically increased in dermal fibroblasts, and PCNA was increased in the neo-epidermis by KY19382 treatment (Figure 4A,B). In addition, the expression level of Keratin 14 was increased, especially in the newly formed epidermis, after treatment with KY19382 (Figure 4A,B).

Activation of the Wnt/β-catenin pathway activates adult stem cells, and this plays a key role in cutaneous wound healing [11,32]. To identify the effect of KY19382 on stem cell activation in the wounds, the expression levels of CD34 and Nestin, the stem cell markers, were detected by IHC analyses. We observed that CD34- or Nestin-positive cells were significantly increased in the dermis layer of mice treated with KY19382 compared to those of the vehicle or EGF-treated group (Figure 4C,D).

Taken together, KY19382 effectively accelerates cutaneous wound healing with the induction of essential wound healing markers together with the activation of stem cells and induction of the Wnt/β-catenin pathway.

### 2.3. KY19382 Enhances Re-Epithelialization and Collagen Deposition at an Early Stage of Cutaneous Wound Healing

Both the migration and proliferation of keratinocytes and fibroblasts play crucial roles in wound healing, as they initiate proliferation and play a role in the repair phase; early induction of the proliferative phase is essential for wound healing, and the expression of wound healing markers during this stage is a determining factor for the quality of wound healing [33]. To characterize the time-dependent wound healing effect of KY19382, we performed in vivo cutaneous wound healing assay using KY19382 or EGF as a control, and harvested wound tissues at 1, 4, 7, and 10 d post wounding [22].

**Figure 5:**
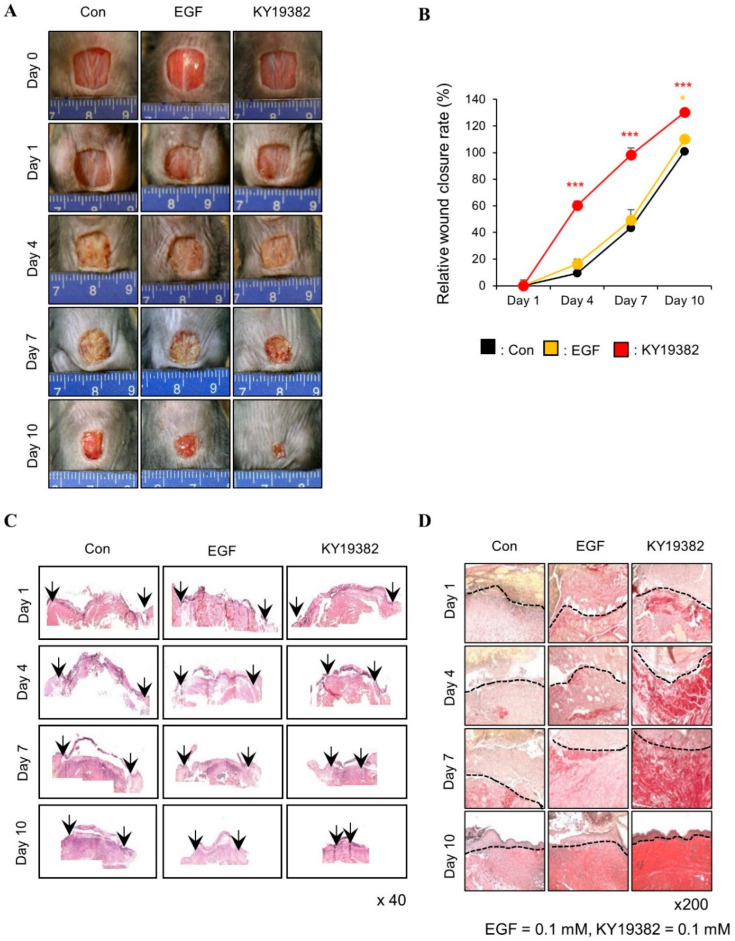
Time-dependent effects of KY19382 in cutaneous wound healing. Full-thickness wounds (diameter = 1.0 cm) were made on the back of 8-week-old male C3H mice, and vehicle, KY19382 (0.1 mM), or EGF (0.1 mM) were topically applied daily on the wounds up to 10 d (*n* = 8 mice/group). Mice were sacrificed at 1, 4, 7, and 10 d post wounding, and wounded tissues excised from each day were subjected to histochemical analyses. (**A**) Representative gross images of wounds at 0, 1, 4, 7, and 10 d post wounding. (**B**) Relative wound closure rates were quantified as percentage of wound closure and closure rates of the control group were considered as 100. Wound sizes were measured every 1, 4, 7, and 10 d after creating the wound. (**C**) Representative images of H&E staining at 1, 4, 7, and 10 d post wounding. Arrows indicate wound edges. (**D**) Representative images of picrosirius red staining of wounded tissues at 1, 4, 7, 10 d post wounding (*n* = 3, independent experiments). Original magnification: (**C**), ×40; (**D**), ×200. Values are expressed as means ± SEM. *, *p* < 0.05; ***, *p* < 0.0005, significantly different from vehicle, control, or as indicated.

The KY19382-treated mice group showed a significant enhancement of wound healing at an early time point, as shown by an advanced wound closure rate and re-epithelialization at 4, 7, and 10 d post wounding compared to those of the vehicle or EGF-treated group (Figure 5A–C). Moreover, collagen deposition at the dermal layer was initiated at an early stage of wound healing in the KY19382-treated group (Figure 5D).

These results show that KY19382 treatment accelerated re-epithelialization and proliferation at an early stage of cutaneous wound healing.

### 2.4. KY19382 Activates Stem Cells and Accelerates Wound Healing at an Early Stage of Cutaneous Wound Healing with the Induction of the Wnt/β-Catenin Pathway

**Figure 6:**
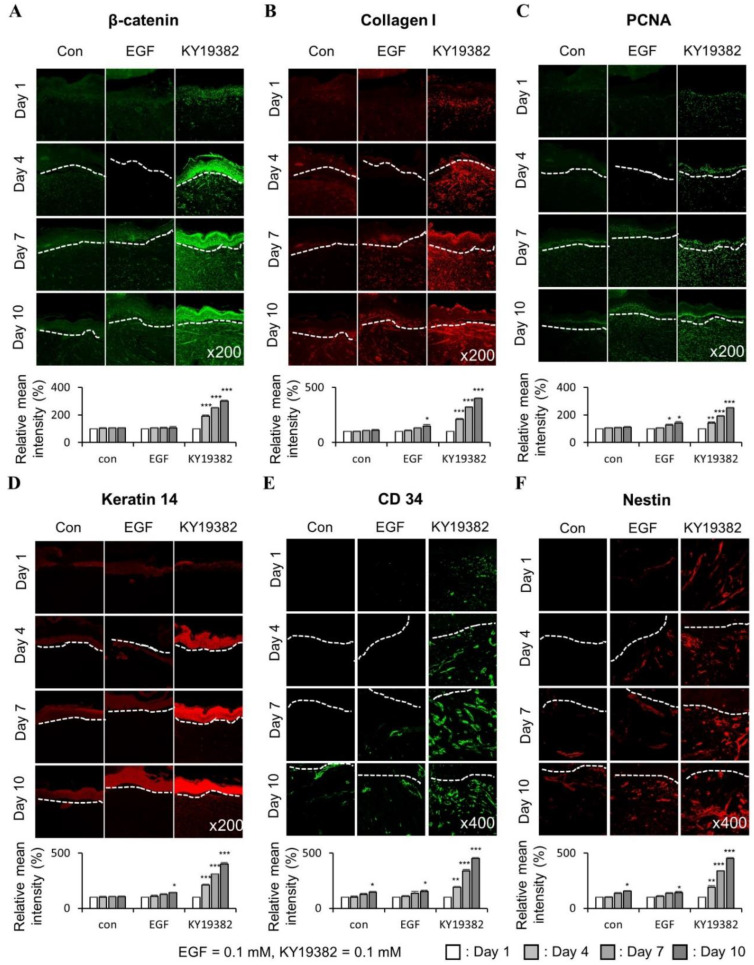
Time-dependent effects of KY19382 in wound healing and stem cell markers. Wounded tissues excised from 1, 4, 7, and 10 d post wounding of Figure 5 were subjected to IHC analyses. (**A**) Representative images for IHC analyses of β-catenin at wounds 1, 4, 7, and 10 d post wounding (**upper**), and quantitative measurements of intensities of β-catenin (**lower**) (*n* = 3 independent experiments). (**B**) Representative images for IHC analyses of Collagen I at wounds 1, 4, 7, and 10 d post wounding (**upper**), and quantitative measurements of intensities of Collagen I (**lower**) (*n* = 3 independent experiments). (**C**) Representative images for IHC analyses of PCNA at wounds 1, 4, 7, and 10 d post wounding (**upper**), and quantitative measurements of intensities of PCNA (**lower**) (*n* = 3 independent experiments). (**D**) Representative images for IHC analyses of Keratin 14 at wounds 1, 4, 7, and 10 d post wounding (**upper**), and quantitative measurements of intensities of Keratin 14 (lower) (*n* = 3 independent experiments). (**E**) Representative images for IHC analyses of CD34 at wounds 1, 4, 7, and 10 d post wounding (**upper**), and quantitative measurements of intensities of CD34 (**lower**) (*n* = 3 independent experiments). (**F**) Representative images for IHC analyses of Nestin at wounds 1, 4, 7, and 10 d post wounding (**upper**), and quantitative measurements of intensities of Nestin (**lower**) (*n* = 3 independent experiments). Dashed lines demarcate the epidermal–dermal boundary. Original magnification: (**A**–**D**), ×200; (**E**,**F**), ×400. Values are expressed as means ± SEM. *, *p* < 0.05; **, *p* < 0.005; ***, *p* < 0.0005, significantly different from vehicle, control, or as indicated.

To further characterize the effects of KY19382 on the profiles of wound healing markers during the process of wound healing, especially at an early stage, tissues obtained from the mice treated with KY19382 at different time points were subjected to IHC analyses. KY19382 treatment significantly induced the protein level of β-catenin in both keratinocytes and fibroblasts of skin tissues at 4, 7, and 10 d post wounding compared to those of vehicle and EGF treatment (Figure 6A). In addition, significant inductions of the protein levels of Collagen I in the dermal layer, PCNA in the neo-epidermis, Keratin 14 in the newly formed epidermis, and stem cell markers, CD34 and Nestin, were observed in mice treated with KY19382 compared with those treated with vehicle or EGF (Figure 6B–F). The increments of the wound healing markers and stem cell markers were correlated with the level of β-catenin by KY19382 treatment (Figure 6).

Overall, these results show that KY19382 accelerates cutaneous wound healing by induction of the wound healing markers with activation of the stem cells at an early stage of the wound healing.

## 3. Discussion

Owing to an expansion of the elderly population, various types of nonhealing wounds, such as chronic wounds, acute wounds, and ulcers, are becoming major threats to public health and the economy [34]. For this reason, there are growing needs for new wound healing agents in medical and cosmetic markets [4].

Current therapies such as those using antibiotics can cause serious organ toxicity and do not enhance the wound healing process itself [5]. The growth factor-based therapies have limitations such as poor absorption, short half-lives, and high cost [6]. The cell-based therapies are considered regenerative and attractive, but have difficulties in the technique, quality control, and application as well as the safety issues [35,36]. Recently, targeting the signaling pathway that regulates tissue regeneration and the wound healing process has emerged as a potential approach for developing wound healing agents [19]. The Wnt/β-catenin pathway is considered a major controller of the wound healing process as it plays an important role in all phases of wound healing, as well as its role of activation of stem cells [7]. The levels of β-catenin and its target genes, such as matrix metalloproteinase 7 and fibronectin, increase in the dermal layer of human wound tissues at the proliferative phase [37]. In addition, the Wnt/β-catenin pathway interacts with other signaling pathways involved in wound healing [20,21]. Therefore, targeting the Wnt/β-catenin pathway is a tempting approach for the development of wound healing agents; however, there is still no such agent available.

In this study, we assessed the effects of KY19382, an activator of Wnt/β-catenin signaling by interference of the role of CXXC5 via inhibiting CXXC5–Dvl PPI, on the enhancement of wound healing in both in vitro and in vivo systems. KY19382 significantly enhanced cell migration and proliferation of human keratinocytes and dermal fibroblasts with an increment of the levels of phalloidin, Keratin 14, PCNA, Collagen I, and α-SMA without causing significant cellular cytotoxicity. We confirmed that the wound healing effects of KY19382 was mediated via the Wnt/β-catenin signaling pathway, as shown by the siRNA-mediated β-catenin knockdown in human keratinocytes and dermal fibroblasts. We also confirmed that KY19382 more effectively accelerated wound healing compared to PTD-DBM, the peptide targeting the same CXXC5–Dvl PPI target, and EGF in a murine acute wound healing model of wound healing, with an increment of the hallmark factors including the collagen deposition in the dermal layer, induction of Keratin 14 in the newly formed epidermis, and PCNA in the neo-epidermis layer with induction of the Wnt/β-catenin signaling pathway. It is known that the CD34-positive cell promotes wound healing via inducing neo-vascularization, and the Nestin-expressing interfollicular blood vessel network contributes to wound healing [38,39]. We confirmed activations of the CD34 and Nestin, the stem cell markers, by KY19382 treatment. No significant stimulation of the stem cell markers was observed by EGF treatment, indicating that the Wnt/β-catenin pathway, but not the EGF-responsive pathways, induces a regenerative effect on the damaged skin.

Moreover, using time-point in vivo wound healing assay, we observed that KY19382 treatment significantly increased the early induction of β-catenin in wounded tissue with stimulation of collagen deposition, re-epithelialization, neo-epidermis formation, and stem cell regeneration at the early stage of wound healing process compared to that of control groups. These results indicate that KY19382 efficiently accelerated cutaneous wound healing at an early stage with induction of the Wnt/β-catenin pathway.

Overall, the small molecule-mediated activation of the Wnt/β-catenin pathway by blockade of the function of the negative feedback regulator CXXC5 could be a highly effective and safe approach for the development of a wound healing agent.

## 4. Materials and Methods

### 4.1. Cell Culture and In Vitro Scratch Assay

The HaCaT human keratinocytes and HDFa human dermal fibroblasts were purchased from Thermo Fisher Scientific (Waltham, MA, USA). Dermal fibroblasts and keratinocytes were cultured using Dulbecco’s modified Eagle’s medium (DMEM; Gibco, Grand Island, NY, USA) contained with 10% (*v*/*v*) heat-inactivated fetal bovine serum (FBS; Gibco), 100 mg/mL of streptomycin (Gibco), and 100 mg/mL of penicillin (Gibco) at 37 °C in a humidified incubator with 5% (*v*/*v*) CO_2_. For in vitro scratch assay, human dermal fibroblasts or HaCaT keratinocytes were seeded in 12-well plates containing 10% FBS DMEM, triplicated at a density of 4 × 10^5^ cells/well. After 24 h of the attachment period, the monolayers of cell surfaces were scratched with a sterile pipette tip and were incubated with a medium containing 5% FBS with or without KY19382 or PTD-DBM (positive control) dissolved in dimethyl sulfoxide (DMSO). After 24 h, cells were harvested, with a washing procedure with cold phosphate buffered saline (PBS, pH 7.4) carried out once, fixed in 4% paraformaldehyde (PFA) for 15 min at room temperature, and were stained with 2% (*w*/*v*) crystal violet. The wound closure area was measured using the NIS-Elements imaging software Ver. 4.50 (Nikon, Tokyo, Japan) (n = 3).

### 4.2. Transwell Migration Assay

Transwell migration assays were performed using matrix-coated transwell plates (8 μm pore size; Corning Life Sciences, Lowell, MA, USA), as described [23]. Filters were coated with bovine serum albumin (BSA) (100 μg/mL) and fibronectin (10 μg/mL) in PBS for 1 h at 37 °C.

Keratinocytes and dermal fibroblasts were seeded onto the filters at a density of 5 × 10^4^ cells/well, and different doses of KY19382 (0.1 or 1 μM) or PTD-DBM (10 μM) dissolved in DMSO were added to the upper and lower compartments before seeding cells. After 24 h of incubation, cells in the inner chamber were removed, and cells on the outer surface were fixed with 4% PFA and stained with 2% (*w*/*v*) crystal violet. Migrated cells were visualized using an optical bright-field microscope (Nikon TE-200U), and the migrated areas were measured using NIS-Elements imaging software (Nikon) (n = 3).

### 4.3. Immunoblot Analysis

Cells and tissues were ground and lysed using RIPA buffer (150 mM NaCl, 10 mM Tris, pH 7.2, 0.1% SDS, 1.0% Triton X-100, 1% sodium deoxycholate, and 5 mM EDTA). Samples were loaded and separated on 10–12% SDS polyacrylamide gels and transferred onto PROTRAN nitrocellulose membranes (Schleicher and Schuell Co., Keene, NH, USA). After blocking with PBS containing 5% non-fat dry skim milk and 0.07% (vol/vol) Tween 20, membranes were incubated with an antibody against β-catenin (1:1000; Santa Cruz Biotechnology, Inc., Dallas, TX, USA), α-smooth muscle actin (1:1000; α-SMA, Abcam, Cambridge, UK), Keratin 14 (1:1000; Covance, Burlington, NC, USA), Collagen I (1:1000; Abcam), proliferating cell nuclear antigen (1:500; PCNA, Santa Cruz Biotechnology), α-tubulin (1:5000; Oncogene Research Products, La Jolla, CA, USA), or Erk (1:5000; Cell Signaling Technology, Beverly, MA, USA) at 4 °C overnight. Samples were then incubated with horseradish peroxidase–conjugated anti-rabbit (1:5000; Bio-Rad Laboratories, Hercules, CA, USA) or anti-mouse IgG secondary antibody (1:5000; Cell Signaling Technology). Protein bands were visualized using an enhanced chemiluminescence kit (Amersham Bioscience, Piscataway, NJ, USA) and a luminescent image analyzer, LAS-4000 (Fujifilm, Tokyo, Japan).

### 4.4. Immunocytochemistry

Keratinocytes and dermal fibroblasts were seeded in 12-well culture plates. Cells were washed once with PBS, fixed with 4% PFA in PBS for 15 min at room temperature, and permeabilized in 0.1% Triton X-100 for 30 min at room temperature. After blocking with 5% BSA for 30 min at room temperature, cells were incubated with primary antibodies against β-catenin (1:100; BD Transduction Laboratories, Lexington, KY, USA), phalloidin (1:200; Molecular Probes, Eugene, OR, USA), or Collagen I (1:100; Abcam) overnight at 4 °C. Cells were washed three times for 10 min for each with PBS and incubated with Alexa Fluor 555-conjugated or Alexa Fluor 488-conjugated IgG secondary antibody (1:400; Molecular Probes, Eugene, OR, USA) for 1 h at room temperature. Then, cells were washed with PBS three times for 10 min for each and counterstained with 4′,6-diamidino-2-phenylindole (DAPI; 1:5000; Boehringer Mannheim, Mannheim, Germany). Finally, cells were washed once with PBS and visualized under a confocal microscope (LSM510 META; Carl Zeiss, Gottingen, Germany).

### 4.5. β-Catenin Knockdown by Small Interfering RNA Transfection

The human β-catenin small interfering RNA (siRNA) target sequences were 5′-GAAACGGCTTTCAGTTGAG-3′ and 5′-AAACTACTGTGGACCACAAGC-3′. β-Catenin siRNA was transfected into keratinocytes or dermal fibroblasts using Lipofectamine Plus reagent (Invitrogen, Carlsbad, CA, USA) at a final concentration of 100 nM. Then, cells were subjected to in vitro wound healing assay with or without KY19382 (1 μM).

### 4.6. Animals and In Vivo Wound Healing Assay

Seven-week-old male C3H mice were purchased from Orient Bio Co. (Gyeonggi-do, Korea), and they were allowed to adapt to their new environment for a week. All procedures were approved and reviewed by the Institutional Animal Care and Use Committee (IACUC) of Yonsei Laboratory Animal Research Center (IACUC-201708-614-01, IACUC-201804-720-03, and IACUC-202101-1204-02). All animals were maintained under a condition of 12 h light/12 h darkness cycle at 22–25 °C in conventional conditions, and fed a standard rodent chow diet and water. Thus, 8-week-old male C3H mice were anesthetized, dorsal hairs were removed by using hair clippers, skins were cleaned with 70% ethanol, and 1.0 cm^2^ of full-thickness dorsal wounds were created on the upper and lower backs of the mice. To determine the therapeutic potential of KY19382 on wound healing, KY19382 (0.05 or 0.1 mM), PTD-DBM (0.1 mM), or epidermal growth factor (EGF; 0.1 mM, for positive control) dissolved in polyethylene glycol 400 (PEG400) (Sigma-Aldrich, St. Louis, MO, USA) were topically applied daily until wound closure (n = 6). To determine the time-dependent effect of KY19382 on wound healing, KY19382 (0.1 mM) or EGF (0.1 mM) dissolved in polyethylene glycol 400 (PEG400) were topically applied daily until 10 d post wounding (n = 8). Each wound size was measured daily, and wound skin tissue samples were harvested at essential time-points, and were evaluated using histochemical analysis.

### 4.7. Immunohistochemical Analysis

Harvested skin tissues were fixed using 4% PFA and tissues embedded in paraffin were sectioned into 4 μm thickness. The slides were deparaffinized using xylene and rehydrated in graded doses of alcohol. The slides were autoclaved in 110 mM sodium citrate buffer for antigen retrieving. Sections were pre-incubated using PBS, then blocked by PBS containing 1% goat serum and 5% BSA for 30 min at room temperature. Tissue sections were then incubated overnight at 4 °C with primary antibodies against β-catenin (1:100; BD Transduction Laboratories), PCNA (1:500; Santa Cruz Biotechnology), Keratin 14 (1:500; Covance), Collagen I (1:100; Abcam), CD34 (1:500; Abcam), or Nestin (1:500; Thermo Fisher Scientific). The sections were rinsed with PBS, incubated with IgG secondary antibody conjugated to Alexa Fluor 488 or Alexa Fluor 555 (1:400; Molecular Probes) for 1 h at room temperature, and counterstained with DAPI (1:5000; Boehringer Mannheim). Fluorescent signals were visualized by the LSM510 META confocal microscope (Carl Zeiss). For hematoxylin and eosin (H&E) staining, the sections were stained by hematoxylin for 5 min and eosin for 1 min. Then, the slides were dehydrated in a graded dose of alcohol series, cleared in xylene, and mounted in Permount (Fisher Scientific, Waltham, MA, USA). H&E-stained tissue sections were visualized using an optical bright-field microscope (Nikon TE-200U).

### 4.8. TOPFLASH Reporter Luciferase Assay

HEK293-TOP cells were seeded at a density of 2.5 × 10^4^ cells/well into a 96-well plate and incubated with DMEM containing 10% FBS for 24 h. Cells were incubated for 24 h with or without KY19382 dissolved in DMSO. All cell lysates were extracted with 25 μL of 1× reporter lysis buffer (Promega, Madison, WI, USA) per well, and luciferase activity was measured by a microplate luminometer (BMG Labtech, Offenburg, Germany).

### 4.9. CellTiter-Glo Luminescent Cell Viability Assay

Keratinocytes and dermal fibroblasts were seeded at a density of 1 × 10^5^ cells/well in a 24-well plate. Cells were then treated with gradient doses of KY19382 dissolved in DMSO for 24 h. Cell viability was assessed using the CellTiter-Glo mixture, as recommended by the supplier. Adenosine triphosphate (ATP) was quantified spectrophotometrically at 560 nm by a microplate luminometer (BMG Labtech).

### 4.10. Statistical Analysis

Statistical analyses were performed using the unpaired two-tailed Student’s *t*-test. Asterisks indicate statistically significant differences (*, *p* < 0.05; **, *p* < 0.005; ***, *p* < 0.0005). Information regarding the statistical details and methods is indicated in the figure legends, tests, or methods.

## Data Availability

All data associated with this study are available from the corresponding author upon reasonable request.

## Supplementary Materials

The supporting information can be downloaded at: https://www.mdpi.com/article/10.3390/ijms241411742/s1.
