# Supplementary material for: KY19382 Accelerates Cutaneous Wound Healing via Activation of the Wnt/β-Catenin Signaling Pathway
Source: Int J Mol Sci. 2023 Jul 21;24(14):11742. doi: 10.3390/ijms241411742 (PMC10380997; doi:10.3390/ijms241411742)
Supplement: Supplementary file 1 [file ijms-24-11742-s001.zip › ijms-2487853-supplementary.pdf]

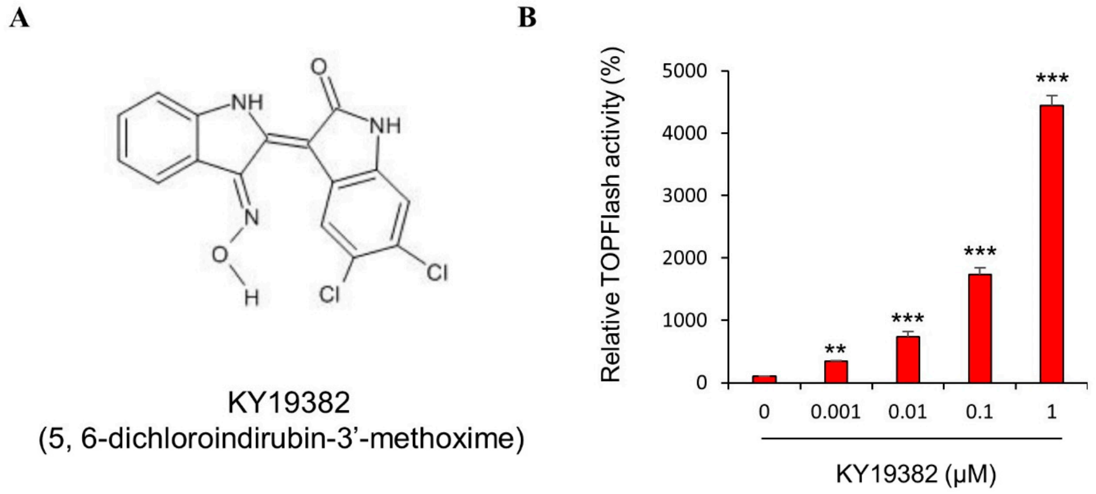

Supplementary figure 1. TOPFlash reporter activity and cellular toxicity tests of KY19382. (A) Structure of KY19382. (B) TOPFlash reporter activity of HEK293 cells treated with vehicle (0.1% DMSO), or shown concentrations of KY19382 for 24 h (*n* = 3). Values are

expressed as means ± SEM. *, P < 0.05; **, P < 0.005; ***, P < 0.0005, significantly different from vehicle, control or as indicated.

A

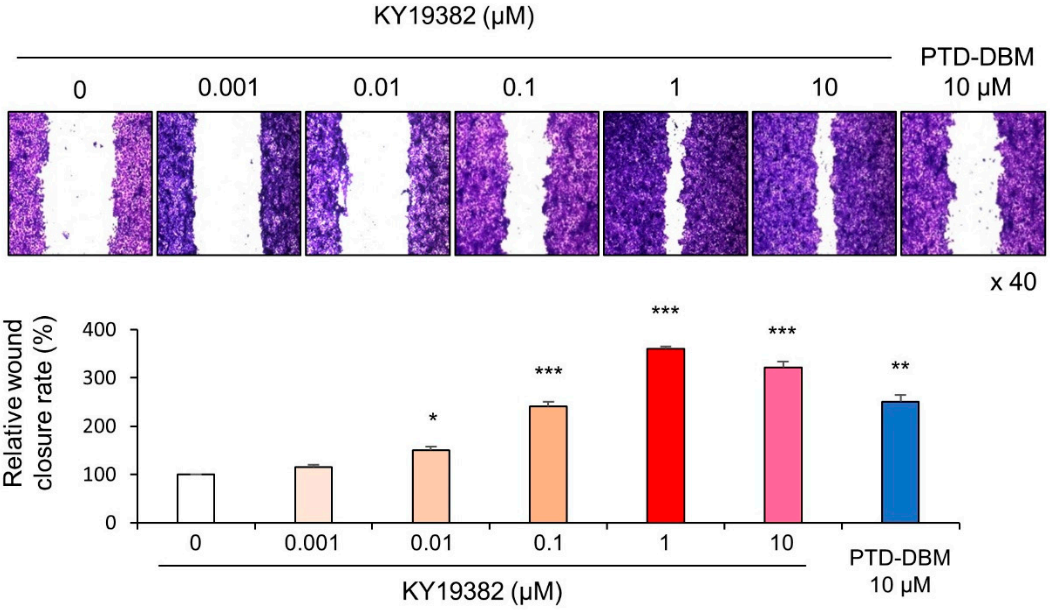

B

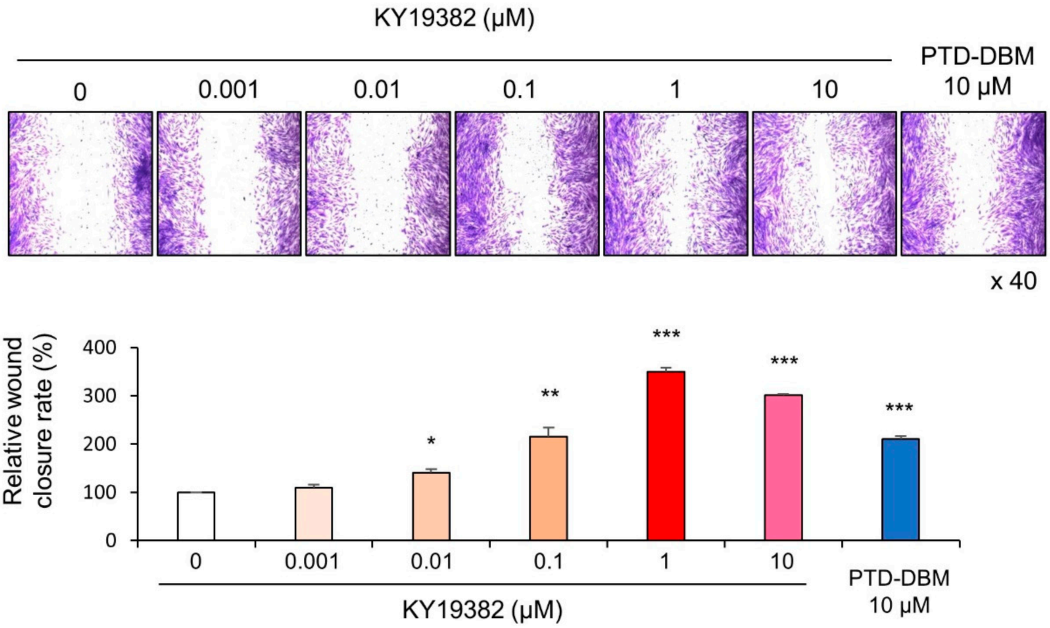

Supplementary figure 2. Dose-dependent effects of KY19382 on migration of human

keratinocytes and dermal fibroblasts. (A, B) Human keratinocytes and dermal fibroblasts were treated with vehicle (0.1% DMSO), or shown concentrations of KY19382 or PTD-DBM (10 μM) for 24 h. (*n* = 3) (A) Representative images of *in vitro* wound healing assay (upper) quantitative measurement of relative wound closure rate (lower) for KY19382 treated human keratinocytes. (B) Representative images of *in vitro* wound healing assay (upper) quantitative measurement of relative wound closure rate (lower) for KY19382 treated human dermal fibroblasts. Values are expressed as means ± SEM. *, $P < 0.05$; **, $P < 0.005$; ***, $P < 0.0005$, significantly different from vehicle, control or as indicated.

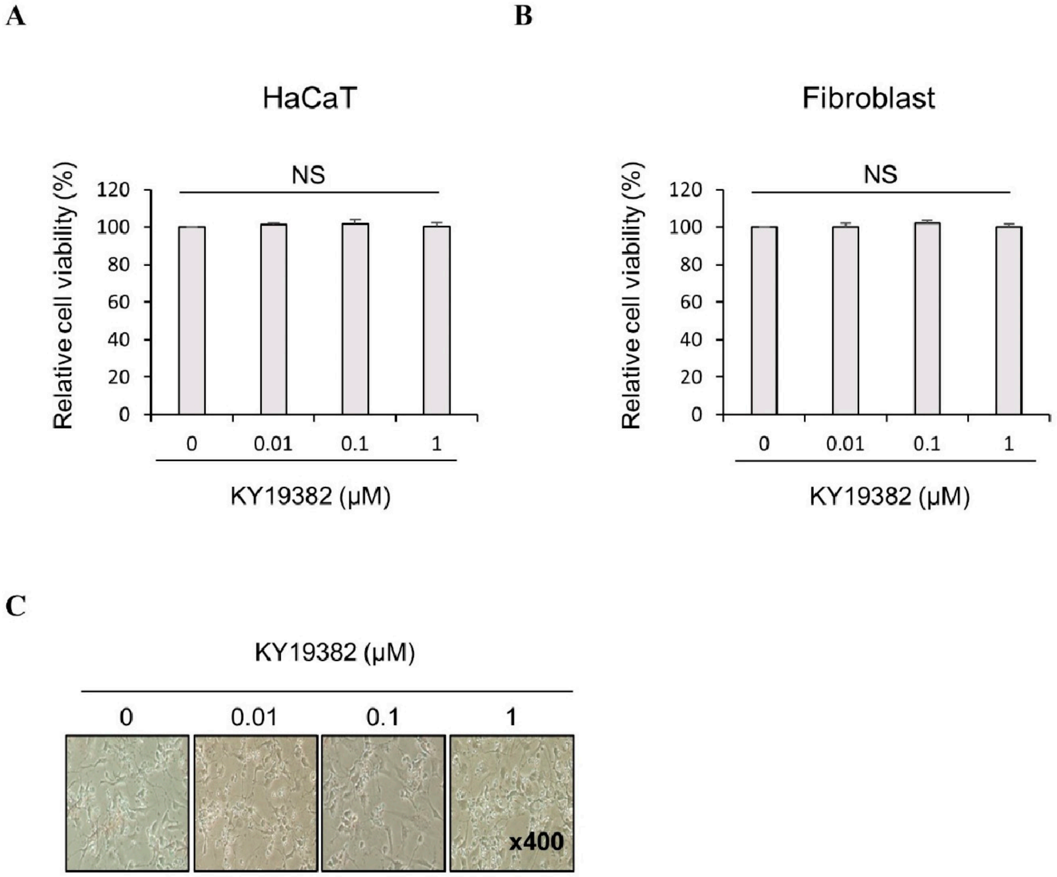

Supplementary figure 3. Cell viability of KY19382 treated keratinocyte, fibroblast, and neural stem cell. (A) Cell viability of HaCaT keratinocytes treated with vehicle (0.1% DMSO), or shown concentration of KY19382 for 24 h (n = 3). (B) Cell viability of human dermal

fibroblasts treated with vehicle (0.1% DMSO), or shown concentrations of KY19382 for 24 h (n = 3). (C) Brightfield images of cultured primary neural stem cell morphology treated by shown concentration of KY19382. Original magnification: (C) x400. Values are expressed as means ± SEM. *, $P < 0.05$; **, $P < 0.005$; ***, $P < 0.0005$, significantly different from vehicle, control or as indicated.
